# Adult-onset primary hemophagocytic syndrome with concurrent Epstein–Barr virus infection: a case report with literature review

**DOI:** 10.3389/fonc.2026.1782084

**Published:** 2026-04-17

**Authors:** Lei Huang, Shuli Guo

**Affiliations:** Department of Hematology, Luoyang Central Hospital Affliated to Zhengzhou University, Luoyang, China

**Keywords:** adult, cryptococcal CNS infection, EBV infection, genetic defects, hemophagocytic syndrome

## Abstract

**Objective:**

To investigate the clinical and laboratory features, phenotypic characteristics, associated genes, and treatment strategies for adult primary hemophagocytic lymphohistiocytosis (HLH).

**Methods:**

The clinical characteristics and underlying causes of an adult primary HLH patient were analyzed according to the HLH-2004 diagnostic criteria. Gene sequences of HLH-related genes (UNC13D, PRF1, STX11, STXBP2, RAB27A, etc.) in the patient and his family were amplified using polymerase chain reaction (PCR) and subsequently analyzed.

**Results:**

The confirmed case involved a 42-year-old male patient presenting with recurrent fever, pancytopenia, hepatosplenomegaly, lymphadenopathy, elevated serum ferritin (600 ng/mL), significantly decreased NK cell activity (3.57%), and elevated soluble CD25 (2777 U/mL), all of which led to a diagnosis of HLH according to the HLH-2004 diagnostic criteria. The patient’s serum EBV-DNA was elevated and was associated with cryptococcal infection of the central nervous system. After antiviral and antifungal treatments, EBV-DNA levels normalized, cerebrospinal fluid examination returned to normal, and primary HLH with an UNC13D gene mutation was confirmed by gene sequencing.

**Conclusions:**

While primary HLH is more prevalent in infants and young children, it can also occur in adolescents and adults, often being misdiagnosed as secondary HLH due to concurrent EBV infection. Molecular genetic alterations are crucial for distinguishing primary from secondary HLH, and HLH-related gene screening remains essential in adult patients.

## Introduction

Hemophagocytic syndrome, also referred to as hemophagocytic lymphohistiocytosis (HLH), encompasses a spectrum of conditions characterized by fever, hepatosplenomegaly, pancytopenia, and hemophagocytosis in the bone marrow, liver, spleen, and lymph nodes. Various pathogenic factors contribute to the abnormal activation and proliferation of lymphocytes, monocytes, and phagocytic cell systems, resulting in the excessive secretion of inflammatory cytokines, which can induce severe and potentially fatal inflammatory states. HLH is classified into two categories: primary and secondary (1). Primary HLH includes: (1) familial HLH (familial hemophagocytic lymphohistiocytosis, FHL), typically occurring in infants and young children; (2) immunodeficiency-related HLH, including Chediak-Higashi syndrome, Griscelli syndrome, Hermansky-Pudlak syndrome II, and others; (3) EBV-associated HLH, such as X-linked lymphoproliferative syndrome (XLP). Among these, familial HLH is the most common, and it is an autosomal recessive genetic disorder. Genetic screening of patients may reveal mutations in genes such as UNC13D, PRF1, STX11, STXBP2, RAB27A, which aid in a definitive diagnosis (2). Secondary HLH results from infections, malignancies, connective tissue diseases, and other underlying conditions.

According to the HLH-2004 diagnostic criteria (3), familial or known genetic defects (including mutations in UNC13D, PRF1, STX11, STXBP2, RAB27A, etc) are identified through molecular biological examination. Alternatively, HLH is confirmed if five of the following eight criteria are met:(1)fever: duration greater than 7 days, with a temperature >38.5 °C; (2) splenomegaly (≥3 cm below the costal margin); (3) Peripheral hemocytopenia (involving two or three cell lines): HGB<90g/L, PLT<100×10^9^/L, neutrophils <1.0×10^9^/L, and not due to decreased hematopoietic function of the bone marrow; (4) Increase in triglycerides and/or decrease in fibrinogen: triglycerides level >3 mmol/L or higher than 3 standard deviations above the reference value for the same age group, and fibrinogen <1.5 g/L or lower than 3 standard deviations below the reference value for the same age group;(5) Hemophagocytes are present in the bone marrow, spleen, or lymph nodes;(6) Decreased or absent natural killer (NK) cell activity; (7) Serum ferritin ≥500μg/L; (8) Elevated soluble IL-2 receptor (sIL-2R or sCD25) levels.

The incidence of primary HLH is approximately 1 in 50,000. More than 90% of cases manifest within the first 2 years of life, with 70% to 80% occurring within the first year; most cases are associated with a positive family history. Adult-onset primary HLH is rarely reported and is often associated with additional precipitating factors, which can result in a high rate of misdiagnosis and missed diagnoses. This paper reports a case of adult HLH with the aim of enhancing clinical understanding of primary HLH among healthcare professionals, improving the screening of suspected cases, and ultimately optimizing the long-term prognosis of patients.

## Case report

**Figure 1:**
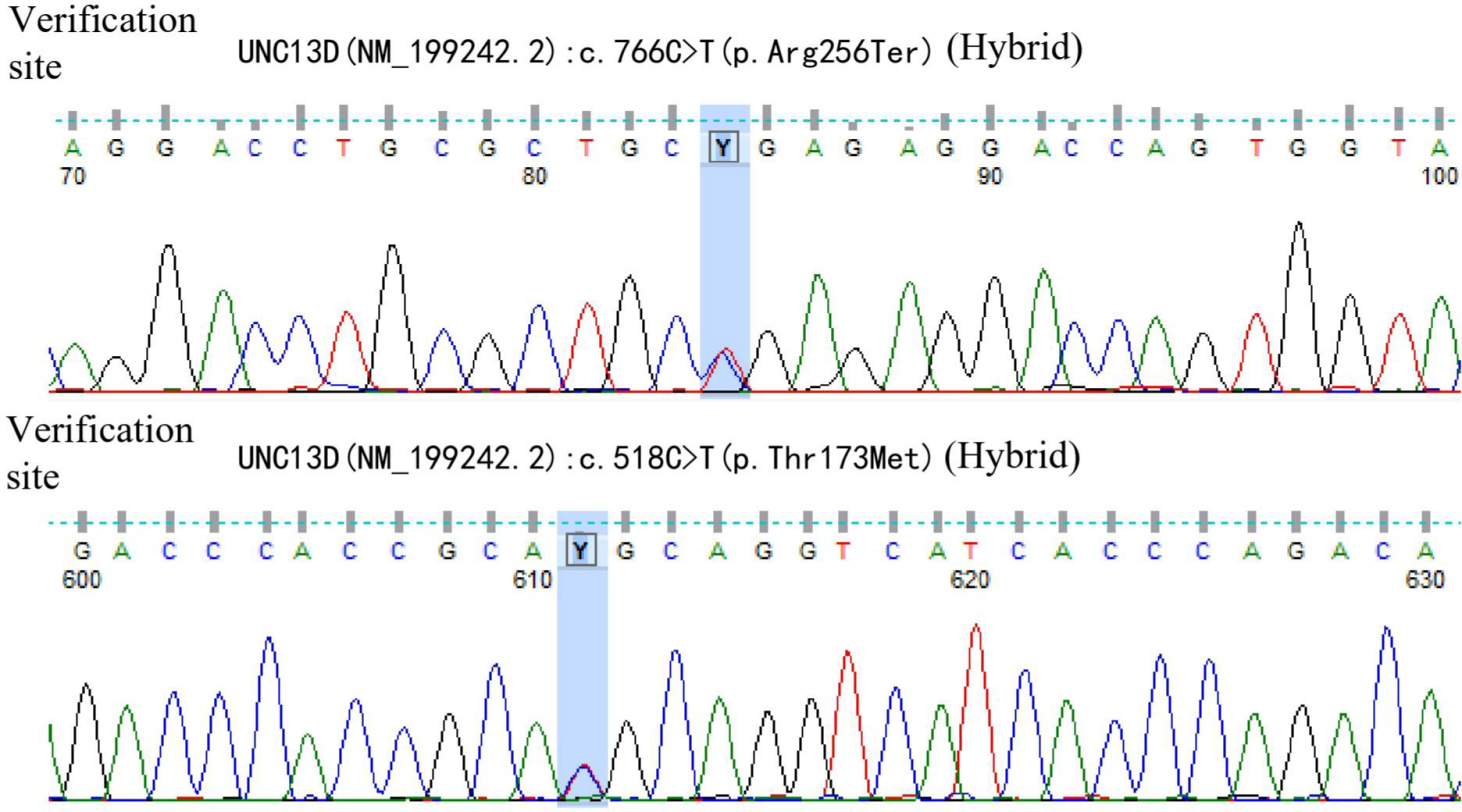
Gene exon sequencing results of the patient.

**Figure 2:**
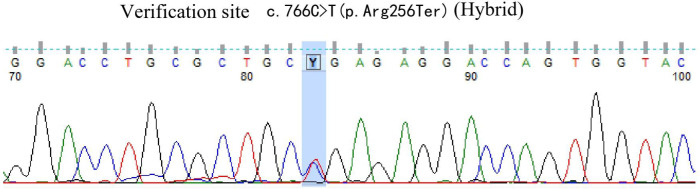
Gene exon sequencing results of the mother.

**Figure 3:**
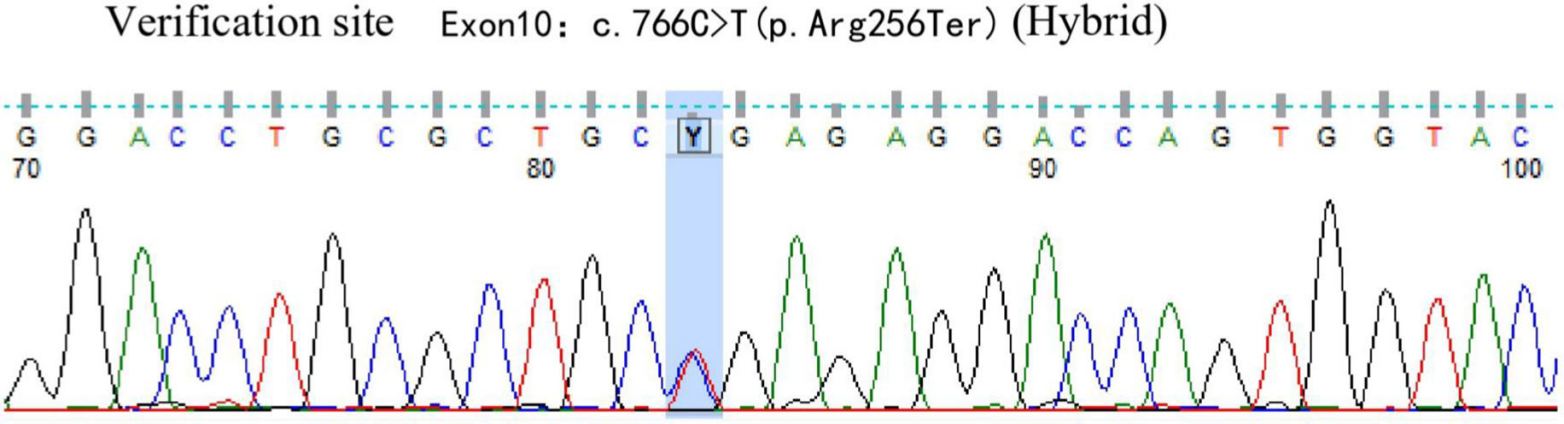
Gene exon sequencing results of the second brother.

**Figure 4:**
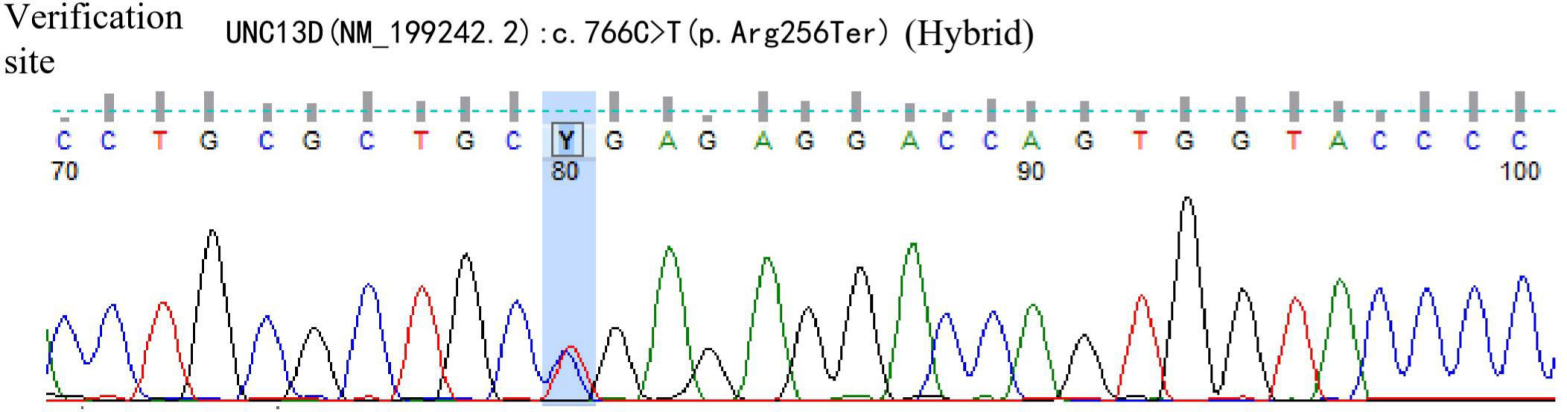
Gene exon sequencing results of the son.

**Figure 5:**
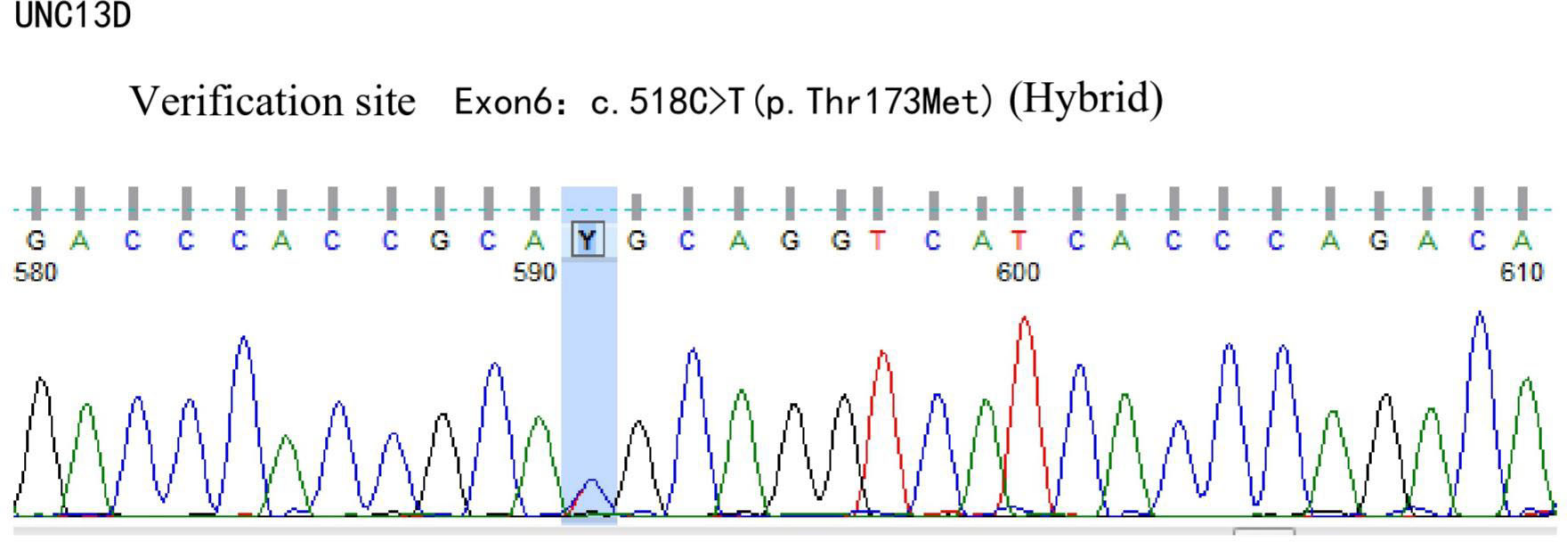
Gene exon sequencing results of the father.

**Figure 6:**
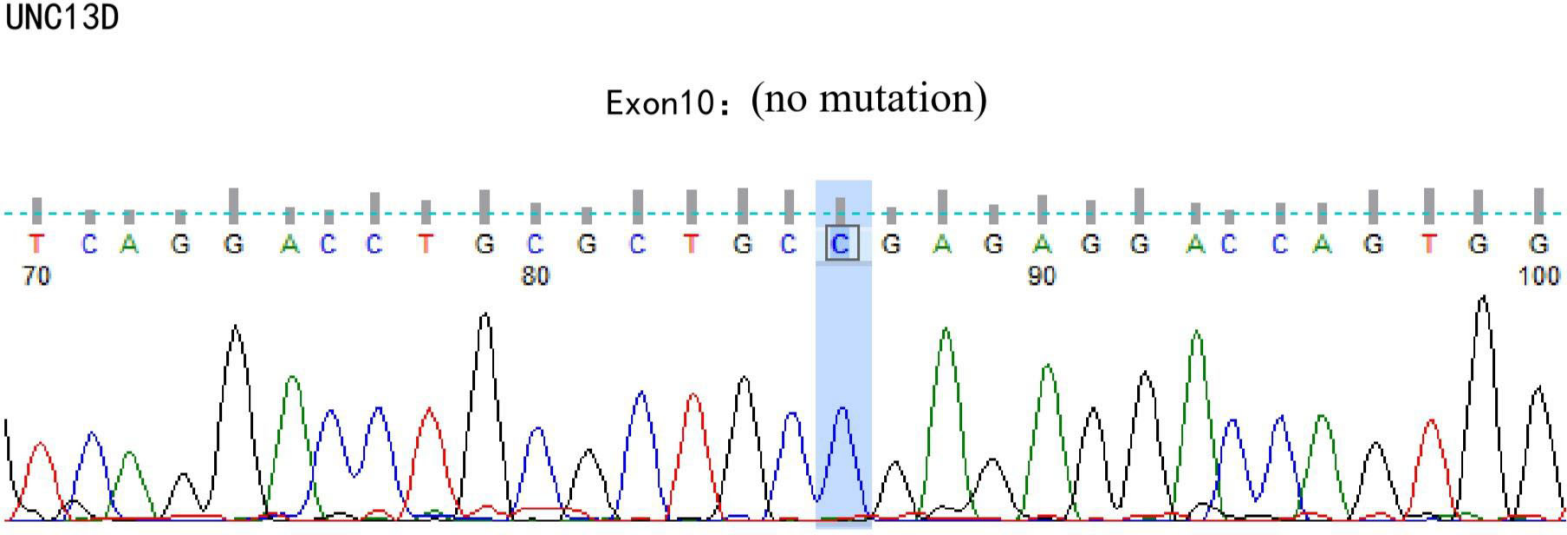
Gene exon sequencing results of the eldest brother.

**Figure 7:**
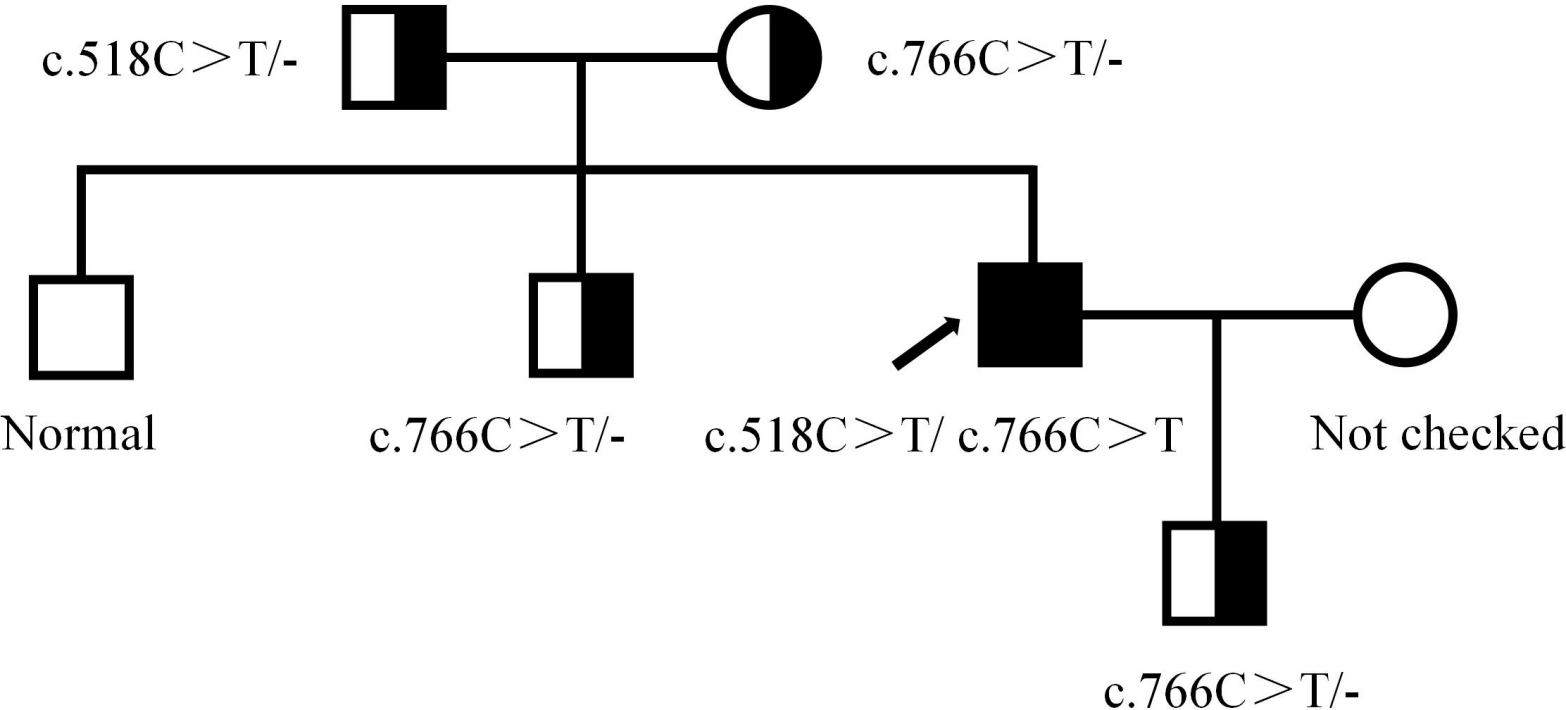
Genealogical tree.

A 42-year-old male patient was hospitalized on February 20th, 2019, with complaints of “intermittent fever for 1 week,” accompanied by chills, headache, dizziness, cough, lower limb pain, and fatigue. Blood test results showed: WBC 5.88×10^9^/L, neutrophils 39.6%, lymphocytes 50.2%, HGB 136 g/L, PLT 88×10^9^/L, CRP within normal range, and negative influenza A/B. Antiviral therapy was ineffective. Two years prior, the patient presented with pancytopenia and splenomegaly. Hepatitis virus tests were negative, and the patient underwent a splenectomy at another hospital (pathological findings: splenic congestion). Following this, liver enlargement was noted, but no further diagnosis was pursued. Upon admission to our hospital, blood tests showed: WBC 3.67×10^9^/L, neutrophils 39.6%, lymphocytes 50.2%, HGB 108 g/L, PLT 63×10^9^/L, LDH 258 U/L, and ferritin 600 ng/mL. EBV antibody was positive, and EBV-DNA levels were significantly elevated upon repeated testing.The cerebrospinal fluid examination suggested a cryptococcal infection(Cryptoccus neoformans, diagnosed by India Ink staining and culture), the opening pressure during lumbar puncture was high. Cryptoccal CNS infection was meningeal involvement. Abdominal CT revealed liver enlargement, pulmonary infection, pleural effusion, pericardial effusion, and multiple retroperitoneal lymph node enlargements. Bone marrow imaging revealed active hyperplasia, with abnormal lymphocytes constituting 9-20%, which were irregular and fusiform in shape. Flow cytometry analysis revealed a population of cells exhibiting the following phenotypes: CD34-, CD117-, CD2+, cCD3+, CD20-, CD19-, CD5-, CD10-, mCD3-, CD4-, CD8-, CD7-, CD56-, CD16-, CD94-, CD30- cells, with a strong expression of CD45, comprising 9.1% of the nuclear cells and displaying large cell bodies. Bone marrow biopsy indicated hyperactive hyperplasia, with an increased proportion of lymphocytes. T-cell receptor (TCR) rearrangement was positive; systemic PET-CT was negative; lymph node biopsy confirmed reactive lymphoproliferative disease, with EBER *in situ* hybridization positive. Re-examination of a spleen biopsy from two years ago revealed significant lymphoid tissue infiltration in the sinus, with the observation of hemophagocytosis. EBER *in situ* hybridization was scattered positive. Soluble CD25 (SCD25) level was elevated at 2777 U/mL, while NK cell activity was decreased at 3.57%. Secondary hemophagocytic lymphohistiocytosis (HLH) induced by Epstein-Barr virus (EBV) infection was diagnosed according to the HLH-2004 diagnostic criteria. Moxifloxacin and cefazoxime were administered for bacterial infection, ganciclovir and peramivir for viral infection, amphotericin B and flucytosine in combination with fluconazole for cryptococcal infection, and chemotherapy (VP-16 combined with dexamethasone) was provided according to the HLH-94 regimen. Although the patient is an adult, given the long history of immune deficiency and the concurrent cryptococcal infection, primary HLH is considered a possible diagnosis. Next-generation HLH-related gene sequencing was performed, and the exonic regions of HLH-related genes were screened for mutations. The results revealed a nonsense mutation in exon 10 of the UNC13D gene (c.766C>T, p.Arg256Ter), with a variation frequency of 51.3%, which may represent a pathogenic site. A missense mutation was identified in exon 6 of the UNC13D gene (c.518C>T, p.Thr173Met), with a variation frequency of 50.33%(PolyPhen-2 HumVar: 0.991, SIFT: 0.00). The clinical significance of this variation remains unclear (**Figure 1**). Primary HLH was confirmed, and mutation screening of the HLH-related gene coding regions was performed in the patient’s family members. The potential pathogenic locus was identified in the mother (**Figure 2**), while the patient’s second brother (**Figure 3**) and son (**Figure 4**) also exhibited the same pathogenic mutation (nonsense mutation in exon 10 of the UNC13D gene: c.766C>T).None of the three pathogenic gene carriers exhibited a similar medical history. The patient’s father carried a missense mutation in exon 6 of the UNC13D gene (**Figure 5**), while the eldest brother (**Figure 6**) did not have any pathogenic gene mutations. This was confirmed as familial HLH (**Figure 7**). Following anti-infective treatments, the symptoms of hemophagocytosis were controlled by the HLH-94 regimen, with improvements in fever and liver enlargement. During outpatient follow-up, the patient was prescribed oral cyclosporine, and routine blood tests stabilized. Lactate dehydrogenase (LDH) and ferritin levels gradually decreased to normal ranges. HLA matching with the eldest brother indicated a half-matched result, and the patient is planning to undergo haplo-identical hematopoietic stem cell transplantation.

## Discussion

There are only a few reports of adult-onset primary hemophagocytic lymphohistiocytosis (HLH). A 48-year-old male patient with primary HLH was reported by Wang Yini et al. (4), who is considered the oldest reported patient in China. A 62-year-old male patient, reported by Nagafuji et al., is believed to be the oldest primary HLH patient worldwide. This paper presents a case of a 42-year-old male patient with primary HLH.

Secondary hemophagocytic lymphohistiocytosis (HLH) is typically induced by infection, tumors, autoimmune diseases, or other factors, and primarily affects adult patients with identifiable underlying conditions. Its clinical characteristics are similar to those of primary HLH. The initial diagnosis of primary HLH is often based on early onset and a positive family history; however, adult primary HLH may be misdiagnosed as secondary HLH. In recent years, advancements in genetic diagnostics have led to the identification of five key familial HLH-related genes (UNC13D, PRF1, STX11, SH2D1A, RAB27A), mutations in which are now recognized as crucial for diagnosis. Based on these findings, the International Association of Tissue Cells developed the HLH-2004 diagnostic guidelines, which emphasize gene defects as essential criteria for distinguishing primary from secondary HLH. Studies by Wang Yini et al. (4), Nagafuji et al. (5), and Sieni et al. (6) have used HLH-related gene mutation detection to confirm cases of adult primary HLH. Specifically, Sieni et al. (6) reported 11 cases of adult primary HLH in patients aged 18 to 43, with a median age of 23, underscoring the importance of HLH-related gene mutation screening for accurate diagnosis and treatment in adult HLH patients.

This patient was initially diagnosed with secondary hemophagocytic lymphohistiocytosis (HLH) due to Epstein-Barr virus (EBV) infection. After anti-infective and anti-cryptococcal treatment, along with chemotherapy, the patient’s symptoms, including fever and liver enlargement, gradually improved. The serum EBV-DNA levels returned to normal, and cerebrospinal fluid examination was also normal. Although the patient is an adult, the long history of immune deficiency and the concurrent cryptococcal infection prompted consideration of primary HLH.

Next-generation sequencing of HLH-related genes was performed. The sequencing results revealed mutations in the UNC13D gene, and the patient was ultimately diagnosed with primary HLH. Additionally, HLH-related gene sequencing was conducted on family members. The pathogenic mutation was likely inherited from the mother, while the patient’s second brother and son also exhibited potential pathogenic mutations in exon 10 of the UNC13D gene. A missense mutation in exon 6 of the UNC13D gene was identified in the father, confirming a diagnosis of familial HLH. These findings underscore the importance of HLH-related gene screening, as molecular genetic mutations are critical for distinguishing primary from secondary HLH.

HLH is associated with a poor prognosis and high mortality. Riviere et al. followed up on 162 adult HLH patients, reporting a mortality rate of 58% (7). Currently, first-line treatments for HLH include the HLH-94 and HLH-04 regimens. The HLH-04 regimen, in comparison to HLH-94, incorporates cyclosporine A (CsA), which reduces T cell activity and modulates the cytokine storm characteristic of HLH. However, due to the patient’s extremely severe condition at the time of onset, as well as concurrent EB virus infection and central nervous system infection, considering the immunosuppressive effects of cyclosporine and its potential impact on liver and kidney function, we choose the HLH-94 protocol. Additionally, literature suggests that the HLH-04 regimen does not offer significant advantages over the HLH-94 regimen (8). The patient’s condition improved following HLH-94 chemotherapy and anti-infection treatment. Cyclosporine was administered orally for maintenance therapy during outpatient follow-up(8 weeks later). As of the most recent follow-up, the patient has normal body temperature, liver volume, and liver function, with normal levels of lactate dehydrogenase (LDH) and ferritin, and overall stable health. When patients meet the clinical diagnostic criteria for HLH, prompt initiation of immunotherapy and chemotherapy, along with enhanced anti-infective and symptomatic supportive care, is crucial for controlling the disease and reducing mortality.

Chemotherapy can reverse the hyperinflammatory state in HLH patients, while hematopoietic stem cell transplantation (HSCT) can restore normal immune function, offering the potential for a cure in most cases. Overall survival (OS) rates can reach 71%, and event-free survival (EFS) rates can reach 60% five years after transplantation (9). For transplantation, the optimal donor is an HLA-matched sibling without HLH-related genetic mutations, followed by a non-consanguineous donor, haploidentical, or half-matched sibling without HLH-related mutations (10). The patient in this case was definitively diagnosed with familial HLH. Although currently stable, the disease remains prone to relapse and can threaten life at any time. Therefore, transplantation should be strongly considered, even with a half-matched sibling donor, when feasible. Transplantation performed during remission significantly improves both the success rate and long-term survival of patients (11).Hematopoietic stem cell transplantation (HSCT) for primary HLH is associated with a high transplant-related mortality rate (30%), primarily due to complications such as infections, hepatic veno-occlusive disease, non-infectious pneumonia, and a high rejection rate (12). Consequently, preventing complications and improving transplant success rates remain significant challenges.

The patient’s father, mother, second brother, and son all carry HLH-related gene mutations, making them unsuitable donors. No matching donor was available in the bone marrow bank. The patient’s eldest brother does not carry any HLH-related gene mutations, and HLA half-matching was confirmed between the patient and the eldest brother, making him the selected donor. Given that the patient’s stable period may be brief, preparations for HLA half-matched hematopoietic stem cell transplantation are currently underway.

Given the rapid progression and high mortality rate of HLH, patients presenting with recurrent fever, liver enlargement, splenomegaly, lymphadenopathy, and pancytopenia must be closely monitored. Diagnostic tests, including ferritin levels, lipid profiles, NK cell function, and CD107a assays, should be conducted promptly to establish an accurate diagnosis and initiate chemotherapy without delay. Additionally, identifying the underlying cause is crucial, and treatment should be tailored accordingly. In adult patients, the possibility of primary HLH cannot be ruled out. For HLH cases with unknown etiology, a prolonged history, or recurrent episodes, gene sequencing should be performed as soon as possible to exclude familial HLH.

## Data Availability

The original contributions presented in the study are included in the article/supplementary material, further inquiries can be directed to the corresponding author/s.
